# Cuproptosis-related immune checkpoint gene signature: Prediction of prognosis and immune response for hepatocellular carcinoma

**DOI:** 10.3389/fgene.2022.1000997

**Published:** 2022-10-05

**Authors:** Tianhao Cong, Yingen Luo, Yu Liu, Chao Yang, Hongcai Yang, Yujie Li, Jingui Li, Xiao Li

**Affiliations:** Department of Interventional Therapy, National Cancer Center/National Clinical Research Center for Cancer/Cancer Hospital, Chinese Academy of Medical Sciences and Peking Union Medical College, Beijing, China

**Keywords:** immune checkpoint, cuproptosis, hepatocellular carcinoma, immune infiltration, prognosis

## Abstract

Immune checkpoint genes (ICGs), the foundation of immunotherapy, are involved in the incidence and progression of hepatocellular carcinoma (HCC). Cuproptosis is characterized by copper-induced cell death, and this novel cell death pathway has piqued the interest of researchers in recent years. It is worth noting that there is little information available in the literature to determine the relationship between cuproptosis and anti-tumor immunity. We identified 39 cuproptosis-related ICGs using ICGs co-expressed with cuproptosis-related genes. A prognostic risk signature was constructed using the Cox regression and the least absolute shrinkage and selection operator analysis methods. The signature was built using the Cancer Genome Atlas (TCGA)-Liver Hepatocellular Carcinoma database. The TCGA and International Cancer Genome Consortium cohorts were classified into two groups; the low- and high-risk groups were determined using a prognostic signature comprised of five genes. The multivariate Cox regression analysis revealed that the signature could independently predict overall survival. Furthermore, the level of immune infiltration analysis revealed the robustness of the prognostic signature-immune cell infiltration relationship observed for Tregs, macrophages, helper T cells, and naive B cells. Both groups showed significant differences in immune checkpoint expression levels. The gene enrichment analysis was used for characterization, and the results revealed that enriching various pathways such as PI3K-AKT-mTOR signaling, glycolysis, Wnt/beta-catenin signaling, and unfolded protein response could potentially influence the prognosis of patients with HCC and the level of immune infiltration. The sensitivity of the two groups of patients to various drug-targeted therapy methods and immunotherapy was analyzed. In conclusion, the findings presented here lay the foundation for developing individualized treatment methods for HCC patients. The findings also revealed that studying the cuproptosis-based pathway can aid in the prognosis of HCC patients. It is also possible that cuproptosis contributes to developing anti-tumor immunity in patients.

## Introduction

Liver cancer is the third leading cause of cancer-related deaths worldwide, with hepatocellular carcinoma (HCC) accounting for 75% of all primary liver cancer cases (Sung et al., 2021). According to the World Health Organization, HCC will cause approximately one million deaths by 2030 (Villanueva, 2019). Surgical and locoregional methods are first-line treatments for early to advanced-stage liver cancer (Llovet et al., 2021a). Systemic therapies treat approximately 50–60% of HCC patients. (Llovet et al., 2018; Llovet et al., 2021b). Systemic therapy has emerged as a standard treatment option for patients with advanced-stage liver cancer. Sorafenib and lenvatinib are used as first-line treatments for HCC patients (median survival: 11–14 months), and cabozantinib and ramucirumab are used as second-line treatments (median survival: 8–11 months) (Llovet et al., 2022). The immune checkpoint inhibitor (ICI)-based immunotherapy has revolutionized HCC treatment, and promising outcomes obtained with nivolumab (anti-PD-1 antibody), tremelimumab (anti-CTLA-4 antibody), athezolizumab (anti-PD-L1 antibody), and bevacizumab (anti-VEGFA antibody) (Sangro et al., 2013; Chiew Woon et al., 2020; Finn et al., 2020). Over 20 phase III trials using ICI combination therapy are currently in progress. (Llovet et al., 2021b). Furthermore, the U.S. Food and Drug Administration has approved pembrolizumab as monotherapy and the combination of nivolumab and ipilimumab as second-line treatment for advanced-stage HCC patients (Zhu et al., 2018; Yau et al., 2020). Many patients do not respond to immune checkpoint blockade (ICB) treatment, which can be attributed to complex pathogenesis, tumor immune microenvironment characteristics of HCC, and tumor heterogeneity (Ribas and Wolchok, 2018; Centanni et al., 2019). Moreover, specific clinical characteristics affect immunotherapy efficacy (Hu et al., 2019; Yu et al., 2021). Therefore, analyzing molecular or gene signatures and particular models can aid in predicting individual responses to immunotherapy. Researchers discovered a link between immune checkpoint genes (ICGs) and cancer onset and progression. It has also been reported that the ICGs may be potential targets for ICB therapy (Liu et al., 2019; Tan et al., 2021; Wu et al., 2021). The analysis of the available clinical information and expression data on the combination of ICGs can aid in identifying targets for personalized therapy and optimizing the existing therapeutic strategies.

Tsvetkov et al. (2022) recently identified a novel copper-induced cell death pathway known as cuproptosis. Cell death is caused by the direct binding of copper to lipoylated components of the tricarboxylic acid (TCA) cycle, resulting in lipoylates protein aggregation, iron-sulfur cluster protein loss, and proteotoxic stress, eventually culminating in cell death (Tsvetkov et al., 2022). The relationship between various cell death mechanisms associated with ferroptosis, pyroptosis, and necroptosis and the tumor immune microenvironment has previously been reported. Complex crosstalk between anti-tumor immune cells has also been observed (Wang et al., 2019; Tang et al., 2020; Xu et al., 2021). However, the relationship between cuproptosis and anti-tumor immunity has yet to be investigated. Studying the co-expression relationship between cuproptosis-related genes (CRGs) and ICGs can help understand the relationship between cuproptosis and anti-tumor immunity.

This study presents the findings from analyzing the expression levels of the cuproptosis-related ICGs, the interaction between the ICGs and the prognosis of HCC patients, and anti-tumor immunity. The enriched signaling pathway and the correlation between the cuproptosis-related ICGs and infiltrated immune cells were studied to understand the underlying mechanisms better. The association between gene signature and systematic therapy, including targeted therapy and immunotherapy, was investigated. The findings could aid in developing individualized HCC treatment.

## Materials and methods

### Data collection

**FIGURE 1:**
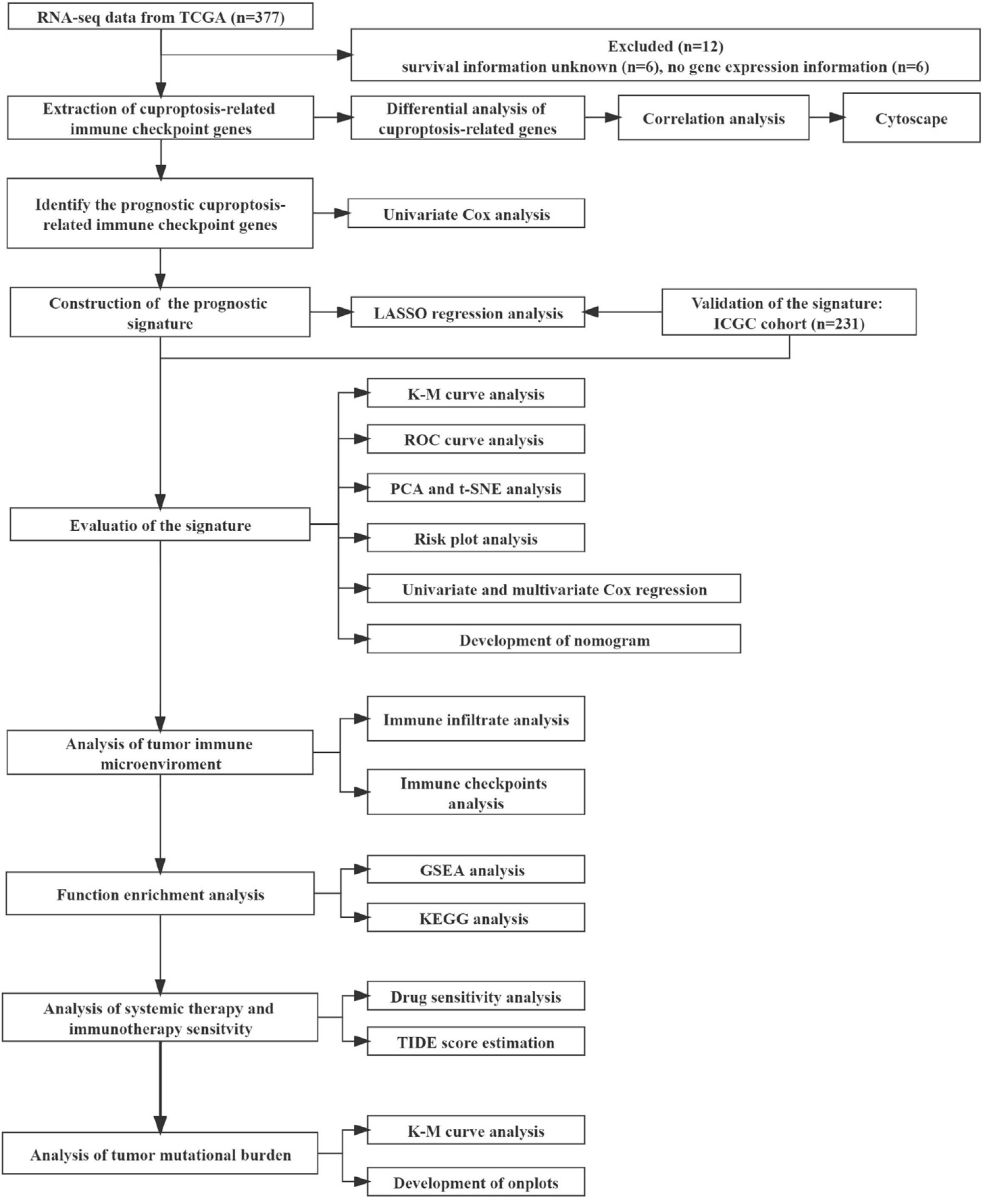
The flowchart of the overall study.

The mRNA expression data were rectified to fragments per kilobase million (FPKM). Data corresponding to simple nucleotide variation and the relevant clinical information of 377 patients were obtained from the Cancer Genome Atlas (TCGA) database (https://portal.gdc.cancer.gov/reposiory). The mRNA expression profiles (normalized read count) and the clinical information of 231 patients were retrieved from the International Cancer Genome Consortium (ICGC) database (https://dcc.icgc.org/projects/LIRI-JP). The gene expression profiles were normalized with the R package “Linear Models for Microarray Data (limma)”. The ICGC and TCGA databases are freely accessible to the public. Therefore, the local ethics committees waived the ethical approval requirement. Several studies (Hu et al., 2020; Oliveri, 2022; Tsvetkov et al., 2022) provided information on the genes linked to ICG and cuproptosis. Finally, 13 CRGs and 79 ICGs were identified and used in the experiments. Our study included 231 HCC patients from the ICGC (LIRI-JP) cohort and 365 HCC patients from the TCGA-LIHC cohort. Data on patients’ clinical baseline characteristics are shown (Table 1). Figure 1 depicts the relevant flow chart.

**TABLE 1:** Clinical baseline characteristics of the patients.

Characteristics	TCGA cohort (*n* = 365)	ICGC cohort (*n* = 231)	Total (*n* = 596)	*p-*value
Gender				0.13
Female	119 (19.97%)	61 (10.23%)	180 (30.20%)	
Male	246 (41.28%)	170 (28.52%)	416 (69.80%)	
Age				
Mean ± SD	59.65 ± 13.36	67.30 ± 10.13	62.61 ± 12.76	
Median [min-max]	61.00 [16.00, 90.00]	69.00 [31.00, 89.00]	64.50 [16.00, 90.00]	
Grade				
1	55 (15.07%)		55 (15.07%)	
2	175 (47.95%)		175 (47.95%)	
3	118 (32.33%)		118 (32.33%)	
4	12 (3.29%)		12 (3.29%)	
NA	5 (1.37%)		5 (1.37%)	
Stage				< 0.05
I	170 (28.52%)	36 (6.04%)	206 (34.56%)	
II	84 (14.09%)	105 (17.62%)	189 (31.71%)	
III	83 (13.93%)	71 (11.91%)	154 (25.84%)	
IV	4 (0.67%)	19 (3.19%)	23 (3.86%)	
NA	24 (4.03%)	0	24 (4.03%)	
Survival Time (days)				
Mean ± SD	811.93 ± 725.80	812.34 ± 418.56	812.09 ± 624.50	
Median [min-max]	596.00 [1.00, 3675.00]	780.00 [10.00, 2160.00]	660.00 [1.00,3 675.00]	
Survival Status				< 0.05
Alive	235 (39.43%)	189 (31.71%)	424 (71.14%)	
Deceased	130 (21.81%)	42 (7.05%)	172 (28.86%)	

### Construction and validation of a prognostic cuproptosis-related immune checkpoint gene signature

The TCGA cohort’s precancerous and cancerous tissues were compared using the “limma” R package to identify the differentially expressed cuproptosis-related genes (DECGs). If the genes met certain criteria (false discovery rate (FDR): < 0.05; |Fold Change|: > 1), they were classified as DECGs. Pearson’s test investigated the correlation between the DECGs and the ICGs (*p* < 0.05; |correlation coefficient|: > 0.21). The differential expression and the correlation analysis were visualized using heatmap and Cytoscape (version 3.6.1). The univariate Cox regression analysis method was used to identify the prognostic genes among the cuproptosis-related immune checkpoint genes (*p* < 0.05). Data overfitting was avoided using the “glmnet” package to integrate the gene expression data, survival status, and survival time. The least absolute shrinkage and selection operator (LASSO)–Cox analysis method was used for regression analysis. A 10-fold cross-validation method was used to construct the optimized model. The risk score was calculated, and the following regression coefficients were determined:

$$\overset{number\;of\;genes}{\underset{x}{\sum }}the\;\;expression\;level\;ofxgene*corresponding\;coeffcient$$

The median risk score was calculated to classify the patients into low- and high-risk groups. The R packages “Survival,” “survminer,” and “timeROC” were used for survival analysis, and the R setting “maxstat” was used to determine the optimal cut-off expression level during the survival analysis of each gene. The “Rtsne” package was used for the t-distributed stochastic neighbor embedding (t-SNE) analysis, and the prcomp function in the “stats” package was used for principal component analysis (PCA). The multivariate Cox regression analysis method (*p*-value < 0.05) was used to identify the independent risk factors. Subsequently, the ICGC database (LIRI-JR) validated the prognostic signature using the same risk score calculation formula and statistical analysis methods. Multivariate and univariate Cox regression analyses were carried out to test the independent prediction ability (*p*-value < 0.05). The “rms” package in R was used to construct a prognostic nomogram, and the calibration curve was used to evaluate the predictive performance of the nomogram. A bootstrap method with 1,000 resamples was used to evaluate the signature’s predictive ability using the concordance index. The TCGA cohort’s results were obtained using multivariate and univariate methods.

### Analysis of the immune infiltration

The relationship between the level of immune infiltration realized and the ICG signature was determined using data from the Tumor Immune Estimation Resource (TIMER 2.0; http://timer.cistrome.org/) and CIBERSORTx (https://cibersortx.stanford.edu). They assess the score of immune infiltrating cells from each TCGA and ICGC cohort sample. The results were obtained for each of the TCGA and ICGC cohort samples. Several methods, including CIBERSORTx algorithm, (Newman et al., 2019), MCPCOUNTER, (Becht et al., 2016), TIMER 2.0, (Li et al., 2016), EPIC, (Racle et al., 2017), xCELL, (Aran et al., 2017), and QUANTISEQ, (Finotello et al., 2019), were used to analyze the relationship between the immune cell infiltration levels and risk scores. The Immuno-Oncology Biological Research (IOBR) package was used to calculate the infiltration scores of immune cells [including macrophages, CD4^+^ T cell, B cell, neutrophils, CD8^+^ T cell, and dendritic cell (DC)]. The expression level of each gene was analyzed to determine the infiltration scores for every patient in the TCGA-LIHC database. Pearson’s correlation coefficient was used to determine the correlation for each signature gene. The “psych” package in R was used to generate the results. Finally, the Wilcoxon test was used to compare the difference in immune checkpoints between the two groups. *P* < 0.05 was accepted as statistically significant.

### Function enrichment analysis

The potential molecular mechanisms and biological functions of the ICG signature were analyzed using the Kyoto Encyclopedia of Genes and Genomes (KEGG) enrichment analysis. The differentially expressed genes (DEGs) for the two groups (high- and low-risk group) were extracted in the limma R package with FDR < 0.05 and |log2FoldChange| >1. The “enrichplot,” “org.Hs.eg.db,” and “clusterProfiler” packages were used for KEGG analysis (statistical significance: *p* and *q* < 0.05). The gene set enrichment analysis (GSEA) method was used to understand the enriched pathways in both groups. The molecular signature dataset was analyzed using Java GSEA v. 4.2.2 and h. all.v7.5.1 symbols. gmt [Hallmarks] (threshold criteria: |NES| > 1 and FDR < 0.05).

### Assessing the clinical significance of the prognostic signature

The “pRRophetic” package was used to determine the drug sensitivity of each sample. The Cancer Genome Project database (https://www.sanger.ac.uk/) was used to analyze the relationship between risk score and drug sensitivity. The analysis was conducted in R using the “pRRophetic” package (*p*-value < 0.05). Simulation studies were conducted using the Tumor Immune Dysfunction and Exclusion (TIDE) algorithm (http://tide.dfci.harvard.edu/) to understand the key mechanisms associated with tumor immune evasion and predict the response potential of tumor immunotherapy. This algorithm simulates the primary mechanisms of tumor immune escape: T cell dysfunction in tumors, cytotoxic T lymphocyte (CTL) invasion levels, and the exclusion properties of T cells in tumors with low levels of CTL invasion (Jiang et al., 2018). The TIDE score, T cell dysfunction, and T cell exclusion were used to estimate immunotherapy efficacy in the TCGA cohort, and the same evaluation was performed in the ICGC cohort for validation. Before TIDE analysis, the gene expression data were normalized.

### Evaluation of genomic features and tumor mutation burden

The single nucleotide variation in the Masked Somatic Mutation type in TCGA-LIHC was downloaded and converted to the mutation annotation format to build waterfall diagrams to visualize gene mutation frequency using the “maftools” package. The differences in TMB realized in the low- and high-risk groups were also studied; *p* < 0.05 was used as the significant difference threshold. The comprehensive survival analysis was carried out based on the TMB level (high- and low-TMB group) and risk score (high- and low-risk group; statistical significance: *p* < 0.05).

### Statistical analysis

The Pearson’s or Spearman’s rank coefficients of correlation were used to investigate the relationship between variables. The continuous variables and normal distributions recorded for the two groups were compared using a t-test or Mann-Whitney U test. The categorical variables were compared using Fisher’s exact or Chi-squared tests. The Kaplan-Meier (K-M) method was used to plot receiver operating characteristic (ROC) curves. The statistical significance was determined using log-rank tests. Using multivariate and univariate Cox regression analyses, independent predictors of overall survival (OS) time were identified. R versions 3.6.1 and 4.1.1 were used to conduct statistical analyses (statistical significance: *p* < 0.05).

## Results

### Identification of prognostic immune checkpoint genes based on differentially expressed cuproptosis-related genes

**FIGURE 2:**
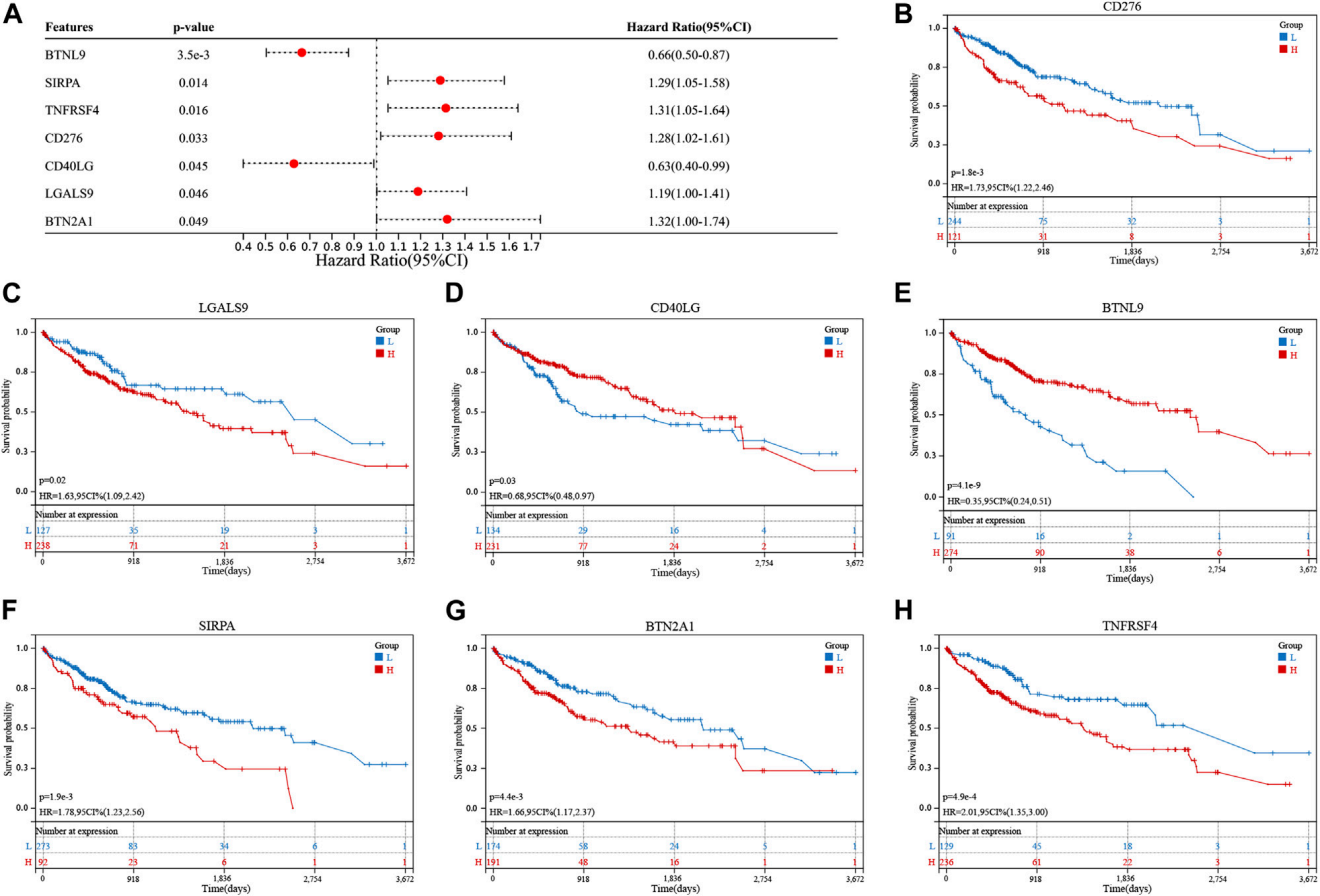
Screening of cuproptosis-related immune checkpoint genes (ICGs) related to the prognosis of hepatocellular carcinoma (HCC) patients. **(A)** Univariate analysis shows that cuproptosis-related ICGs, including *CD276*, *LGALS9*, *CD40LG*, *BTNL9*, *SIRPA*, *BTN2A1*, and *TNFRSF4* genes, were associated with the prognosis of HCC patients. **(B–H)** Kaplan-Meier analysis of cuproptosis-related ICGs related to prognosis.

Differential expression of 11 genes associated with cuproptosis was observed in the TCGA cohort. Eight of these genes were upregulated, and three were downregulated (Supplementary Figure S1A). Thirty-nine immune checkpoint genes were correlated with DECGs (Supplementary Figure S1B). Seven genes related to prognosis were screened out of the 39 genes using the univariate Cox regression analysis method (Figure 2A). The K-M analysis method was used to assess the prognostic significance of the expression levels of these seven genes. High levels of *CD276*, *LGALS9*, *SIRPA*, *BTN2A1*, and *TNFRSF4* genes and low levels of *CD40LG* and *BTNL9* genes reflected poor patient prognosis (Figures 2B–H).

### Prognostic signature construction

**FIGURE 3:**
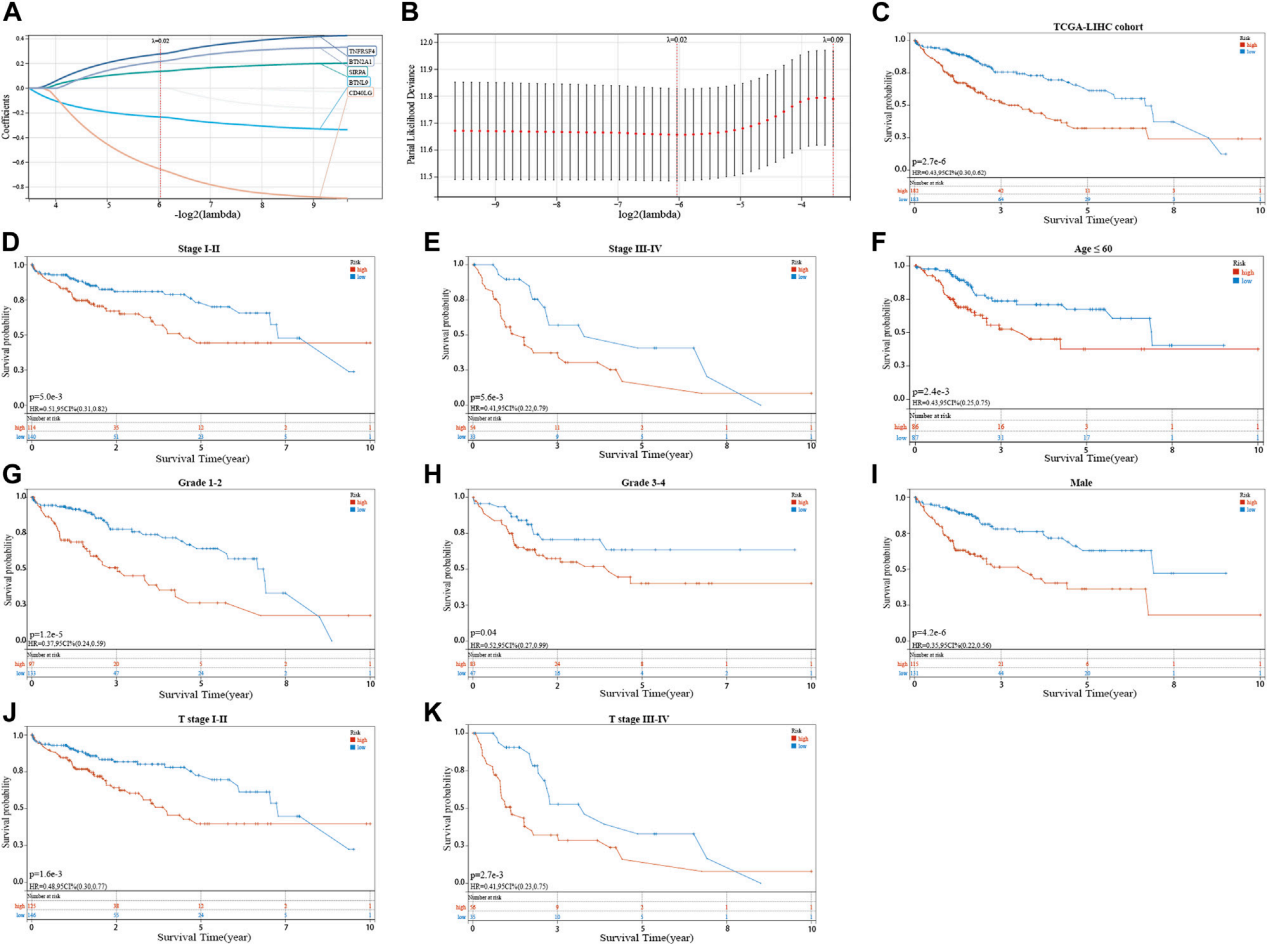
Identifying a prognostic signature based on five cuproptosis-related immune checkpoint genes (ICGs) and their prognostic value. **(A,B)** LASSO Cox regression with 10% discount cross-validation for developing a prognostic signature. **(C)** Kaplan-Meier (K–M) analysis of high- and low-risk groups in the TCGA cohort. **(D–K)** K–M analysis of high- and low-risk groups in different subgroups of the TCGA cohort.

**FIGURE 4:**
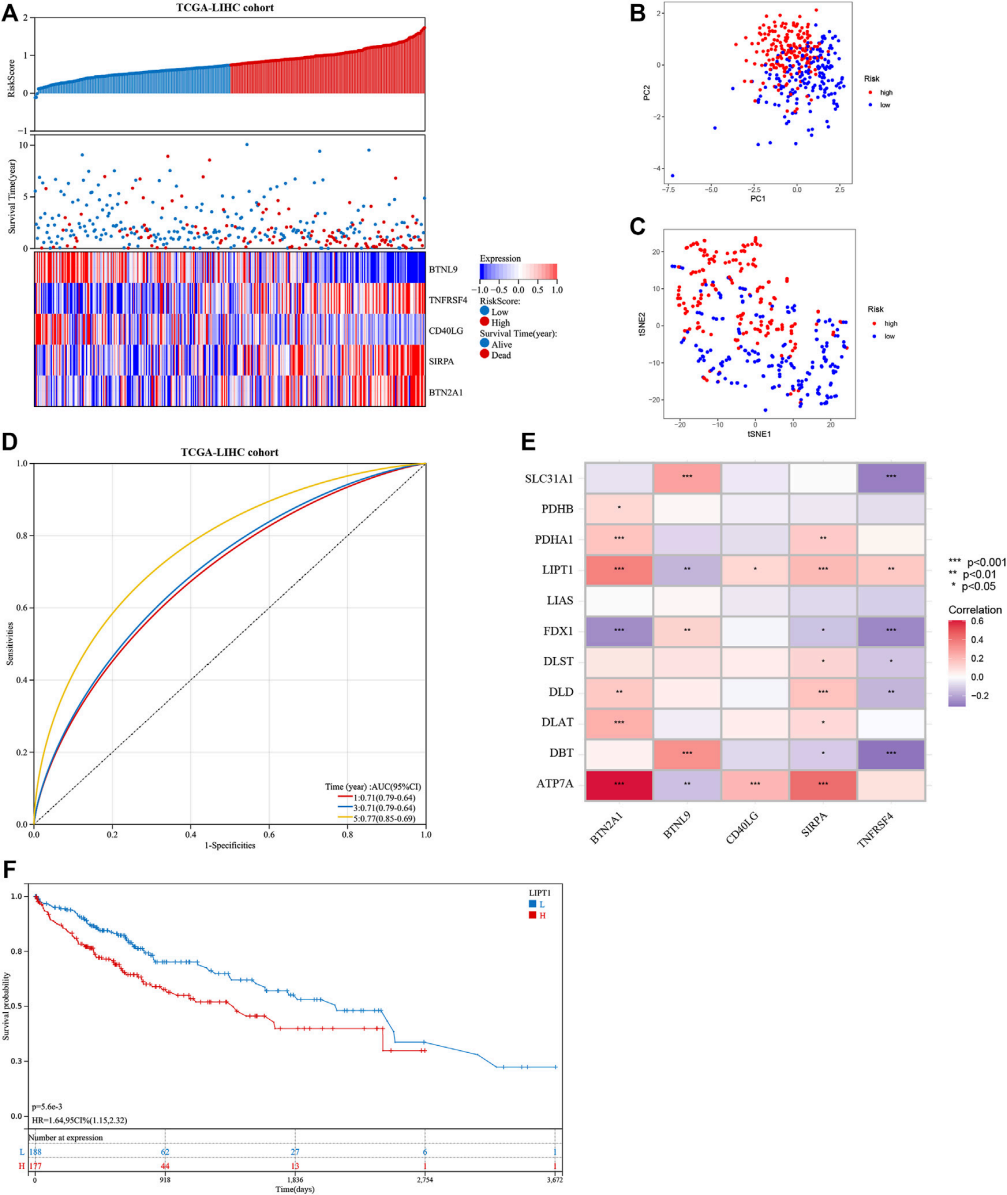
Evaluation of the prognostic signature in the TCGA cohort and the expression of cuproptosis-related genes. **(A)** The distribution of the patient’s risk scores, survival status, and the expression of signature genes for high- and low-risk groups in the TCGA cohort. **(B)** and **(C)** The principal component analysis and t-distributed stochastic neighbor embedding analysis of patients in different risk groups in the TCGA cohort. **(D)** The receiver operating characteristic curves of the prognostic signature for predicting 1-, 3-, and 5-year survival rates of patients in the TCGA cohort. **(E)** Heat map showing the co-expression relationship of differentially expressed cuproptosis-related genes and signature genes. **(F)** Kaplan-Meier analysis of the prognostic value of the *LIPT1* gene.

The seven prognostic gene-based prognostic signature was developed using LASSO-Cox regression analysis. The optimal λ value was used to determine the prognostic signature of five genes (*BTNL9*, *SIRPA*, *TNFRSF4*, *CD40LG*, and *BTN2A1*) (Figures 3A,B). Each sample’s risk score was calculated using the risk score equation (Risk Score = -0.233700910650553 * BTNL9 + 0.137008446999748 * *SIRPA* + 0.278119160353374 * *TNFRSF4*—0.656319252714311 * C*D40LG* + 0.217796049191113 * *BTN2A1*). Based on the median cut-off value, the TCGA cohort was divided into two groups (low-risk: *n* = 183; high-risk: *n* = 182). Figure 3C presents the significant differences in overall survival (OS) between the two groups (*p* < 0.001). K-M analysis was conducted on patients belonging to different subgroups (stages: I–II and III–IV; grades: 1–2 and 3–4; T stages: I–II and III–IV, age ≤ 60)*.* The results indicated that the signature could distinguish between high- and low-risk groups in different subgroups (*p* < 0.05, Figures 3D–K). The prognostic curve, risk plot, and heatmap show the relationship between risk score, survival status, and signature gene expression (Figure 4A). The heatmap presenting the expression profiles of signature genes was scrutinized. The results showed that the expression levels of *TNFRSF4*, *SIRPA*, and *BTN2A1* genes in the high-risk group were higher than those in the low-risk group. It was also observed that the expression levels of *BTNL9* and *CD40LG* genes in the low-risk group were higher than those in the high-risk group. The t-SNE analysis and PCA methods yielded a two-way distribution for patients in different risk groups (Figures 4B,C). The accuracy of the risk scores was determined by analyzing the time-dependent ROC curves (Figure 4D). For the TCGA cohort, the area under curves (AUC) associated with the survival rates were calculated (1-year: 0.71; 3-year: 0.71; and 5-year: 0.77).

The correlation heatmap was constructed, and the relationship between signature genes and DECGs was examined. As shown in Figure 4E, all the signature genes had a significant correlation with *LIPT1* gene expression (*p* < 0.05), and high levels of the *LIPT1* gene were associated with a poor prognosis (*p* < 0.05; Figure 4F).

### Validating the 5-gene signature prognostic value

**FIGURE 5:**
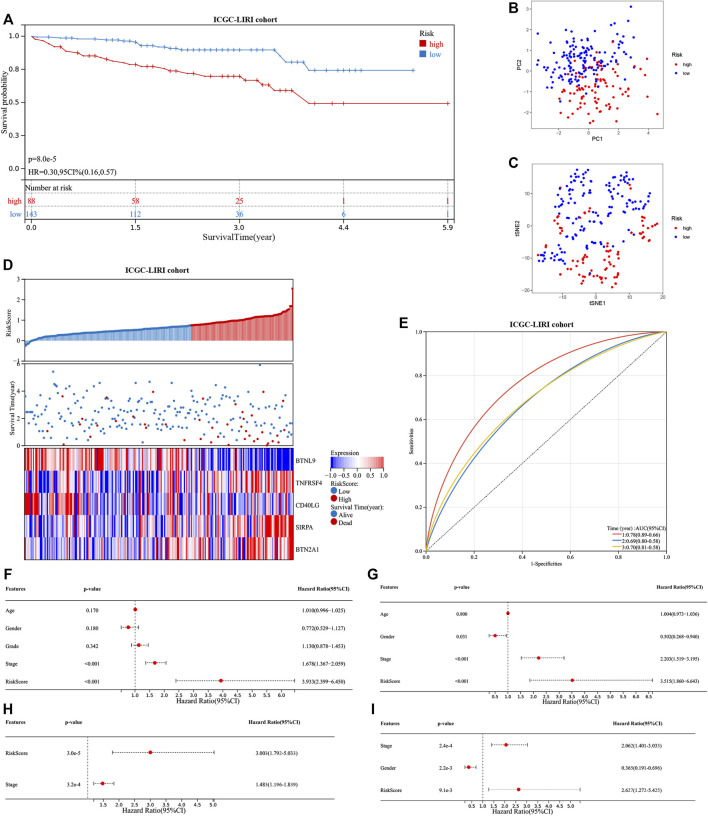
Evaluation of the prognostic signature in the ICGC cohort and the prognostic value in two cohorts. **(A)** Kaplan-Meier analysis of high- and low-risk groups in ICGC cohort. **(B)** and **(C)** The principal component analysis and t-distributed stochastic neighbor embedding analysis of patients in different risk groups, in the ICGC cohort. **(D)** The distribution of the patient’s risk scores, survival status, and the expression of signature genes for high- and low-risk groups in the ICGC cohort. **(E)** The receiver operating characteristic curves of the prognostic signature for predicting 1-, 2-, and 3-year survival rates of patients in the ICGC cohort. Univariate and multivariate Cox regression analyses show the risk score as an independent prognostic factor **(F)** and **(H)** in the TCGA cohort and **(G)** and **(I)** in the ICGC cohort.

The ICGC cohort samples were scored using the same method as the TCGA cohort samples, and the high- and low-risk samples were then categorized using the TCGA cohort’s median cut-off value. The K-M analysis revealed a significant difference in OS between low-risk and high-risk groups (*p* < 0.001, Figure 5A). A significant difference in OS was also observed when the K-M analysis method was used to analyze the various subgroups of the ICGC cohort. Gender, age, and tumor stage of the patients were investigated in the low- and high-risk groups (*p* < 0.05, Supplementary Figures S2A–F). PCA and t-SNE analysis for the ICGC cohort also showed a two-way distribution of patients into two groups (Figures 5B,C). The prognostic curve, risk plot, and heatmap analysis revealed that the number of deaths in the high-risk group was significantly higher, and the expression of signature genes observed in the two groups was comparable to that obtained from the TCGA database (Figure 5D). The AUC values for the ICGC cohort were recorded (1-year: 0.78; 2-year: 0.69; and 3-year: 0.70) (Figure 5E). In addition, the patient’s baseline characteristics were determined and compared (Tables 2,3). Patients in the high-risk group were more likely to be at an advanced stage (*p* < 0.01).

**TABLE 2:** Clinical baseline characteristics of patients with different risk groups in the TCGA cohort.

Characteristics	Low-risk (*n* = 183)	High-risk (*n* = 182)	Total (*n* = 365)	*p*-value
Age				1
> 60	96 (26.30%)	96 (26.30%)	192 (52.60%)	
≤ 60	87 (23.84%)	86 (23.56%)	173 (47.40%)	
Gender				0.11
Female	52 (14.25%)	67 (18.36%)	119 (32.60%)	
Male	131 (35.89%)	115 (31.51%)	246 (67.40%)	
Grade				< 0.01
Grade 1–2	133 (36.44%)	97 (26.58%)	230 (63.01%)	
Grade 3–4	47 (12.88%)	83 (22.74%)	130 (35.62%)	
NA	3 (0.82%)	2 (0.55%)	5 (1.37%)	
Stage				< 0.01
I–II	140 (38.36%)	114 (31.23%)	254 (69.59%)	
III–IV	33 (9.04%)	54 (14.79%)	87 (23.84%)	
NA	10 (2.74%)	14 (3.84%)	24 (6.58%)	

**TABLE 3:** Clinical baseline characteristics of patients with different risk groups in the ICGC cohort.

Characteristics	Low-risk (*n* = 143)	High-risk (*n* = 88)	Total (*n* = 231)	*p*-value
Gender				0.59
Female	40 (17.32%)	21 (9.09%)	61 (26.41%)	
Male	103 (44.59%)	67 (29.00%)	170 (73.59%)	
Age				1
> 60	113 (48.92%)	69 (29.87%)	182 (78.79%)	
≤60	30 (12.99%)	19 (8.23%)	49 (21.21%)	
Stage				< 0.001
I–II	106 (45.89%)	35 (15.15%)	141 (61.04%)	
III–IV	37 (16.02%)	53 (22.94%)	90 (38.96%)	

Multivariate and univariate Cox regression analyses were conducted to determine if our signature is independent of other clinical parameters. The TCGA cohort was studied using the univariate Cox regression analysis method. Correlation between the OS and the risk score was recorded (*p* < 0.001; hazard ratio (HR) = 3.933; 95% confidence interval (CI) = 2.399–6.450) (Figure 5F). The multivariate Cox regression analysis method revealed that the signature was an independent predictor of survival (*p <* 0.001; HR = 3.003; 95% CI = 1.792–5.033) (Figure 5H). Similarly, the ICGC cohort was studied. The univariate (*p <* 0.001; HR = 3.515; 95%; CI = 1.860–6.643) (Figure 5G) and multivariate Cox regression analyses (*p <* 0.01; HR = 2.627; 95% CI = 1.272–5.425) (Figure 5I) results were used to determine the nature of the signature. Our signature was found to be an independent predictor of OS. Further, ROC curves were generated using a combination of the data on stage and the risk score to determine sensitivity and specificity. The value of AUC was increased in the TCGA and ICGC cohorts (Supplementary Figures S3A,B). The construction of the nomogram also confirmed the result, with the risk score having the most weight in the nomogram that predicts the 1-, 3-, and 5-year survival rates (Supplementary Figures S3C,D). The concordance index further confirmed the signature’s predictive ability (Supplementary Figure S3E).

### Immune infiltrate analysis

**FIGURE 6:**
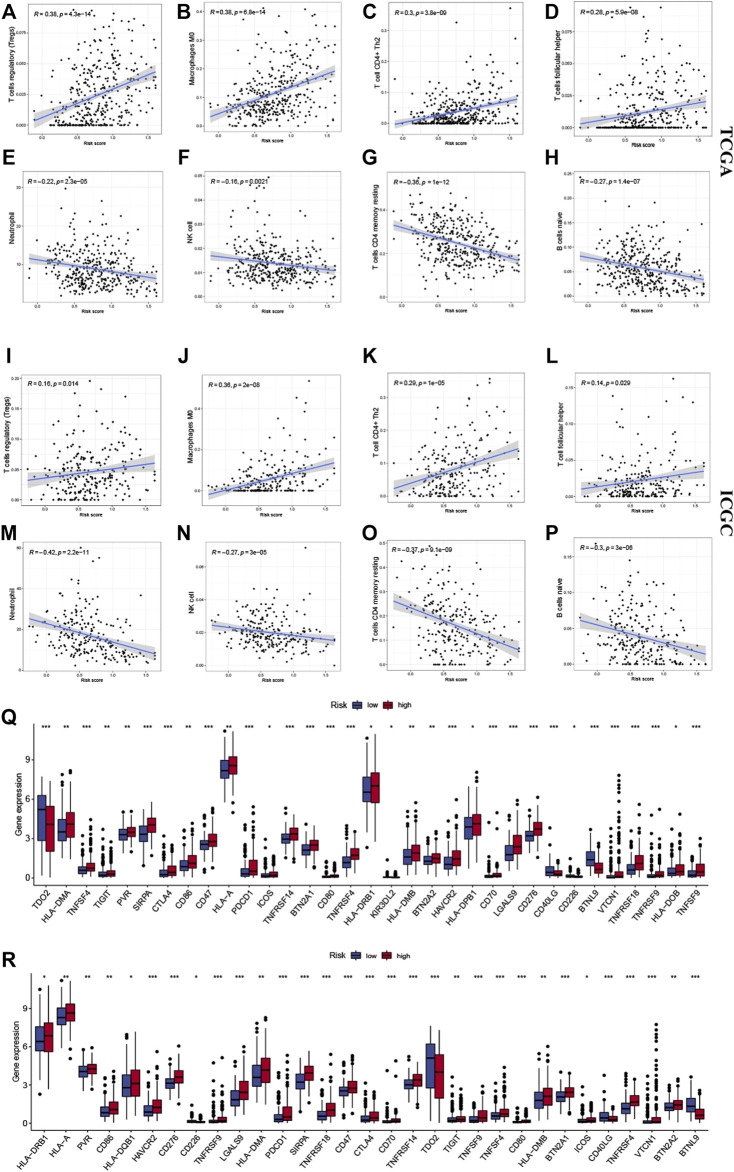
Correlation between the signature and the immune microenvironment. The infiltration of **(A)** Tregs cells, **(B)** M0 macrophages, **(C)** Th2 cells, and **(D)** follicular helper T cells were positively related to risk scores in the TCGA and **(I–L)** ICGC cohorts, respectively. The infiltration of **(E)** neutrophils, **(F)** NK cells, **(G)** memory resting CD4^+^ T cells, and **(H)** naive B cells were negatively related to risk scores in the TCGA and ICGC **(M–P)** cohorts, respectively. The expression of immune checkpoints in high- and low-risk groups, in **(Q)** TCGA and **(R)** ICGC cohorts. The upper and lower ends of the boxes indicate the interquartile range. Lines in the boxes indicate median values, and black dots show outliers. * *p* < 0.05; ** *p* < 0.01; *** *p* < 0.001; ns, no statistical significance.

**FIGURE 7:**
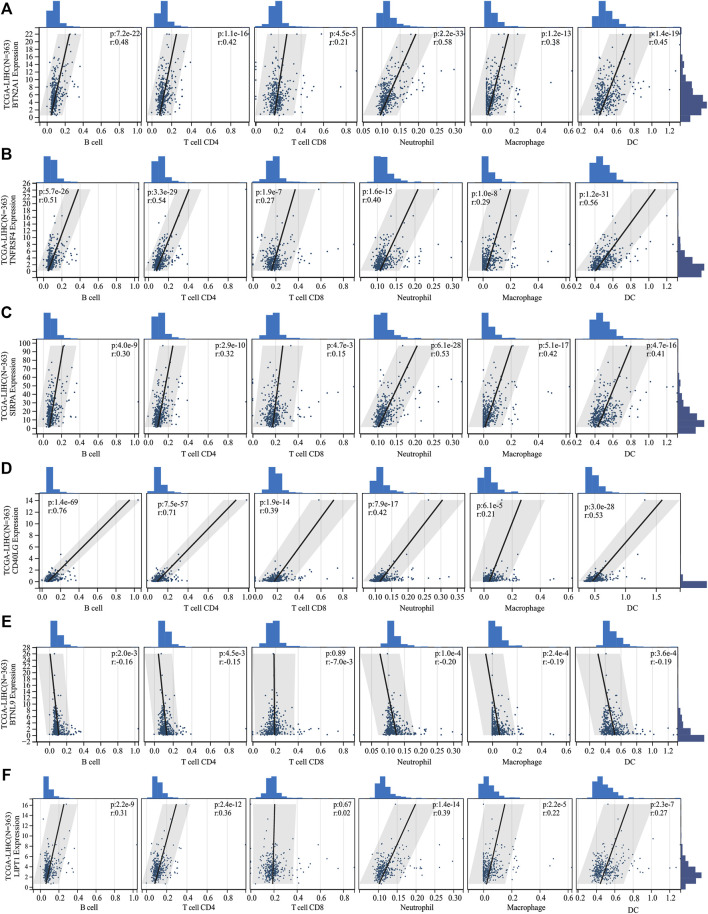
The correlation between the expression of signature genes and immune cell infiltration. The expression of **(A)** *BTN2A1*, **(B)** *TNFRSF4*, **(C)** *SIRPA*, **(D)** *CD40LG*, and **(F)** *LIPT1* genes was positively correlated with immune cell infiltration. The expression of the **(E)** *BTNL9* gene was negatively correlated with immune cell infiltration.

Several algorithms were used to investigate the association between immune cell infiltration level and the signature (Figures 6A–H). The infiltration of Treg cells, M0 macrophages, type 2 helper T cells (Th2 cells), and follicular helper T cells were positively correlated with the risk score (*p* < 0.05). A negative correlation was observed for the infiltration levels of neutrophils, NK cells, memory resting CD4^+^ T cells, and naive B cells (*p* < 0.05). The ICGC cohort’s results were verified under the same algorithms (Figures 6I–P). The results of immune infiltration analysis obtained by different algorithms are summarized in Supplementary Figures S4A,B. Given the clinical significance of immune checkpoint blockade-based immune therapy in HCC, the correlation between the immune checkpoints and the risk score was investigated further. In the TCGA cohort, the high-risk group had significantly higher *PDCD1* and *CTLA4* gene expression levels than the low-risk group (*p* < 0.05; Figure 6Q). The ICGC cohort was analyzed, yielding similar results (Figure 6R). The relationship between the levels of expression of each signature gene and the level of immune cell infiltration was analyzed further. Figure 7 presents a positive correlation between the levels of expression of *BTN2A1, TNFRSF4, SIRPA, CD40LG* genes*,* and immune cells infiltration levels, while the *BTNL9* gene expression level was negatively correlated (Figures 7A–E). Furthermore, the expression levels of the *LIPT1* gene were positively correlated with immune cell infiltration (Figure 7F). These findings demonstrated the prognostic signature’s robustness and association with the tumor immune cell infiltration level.

### Function enrichment analysis based on the prognostic signature

**FIGURE 8:**
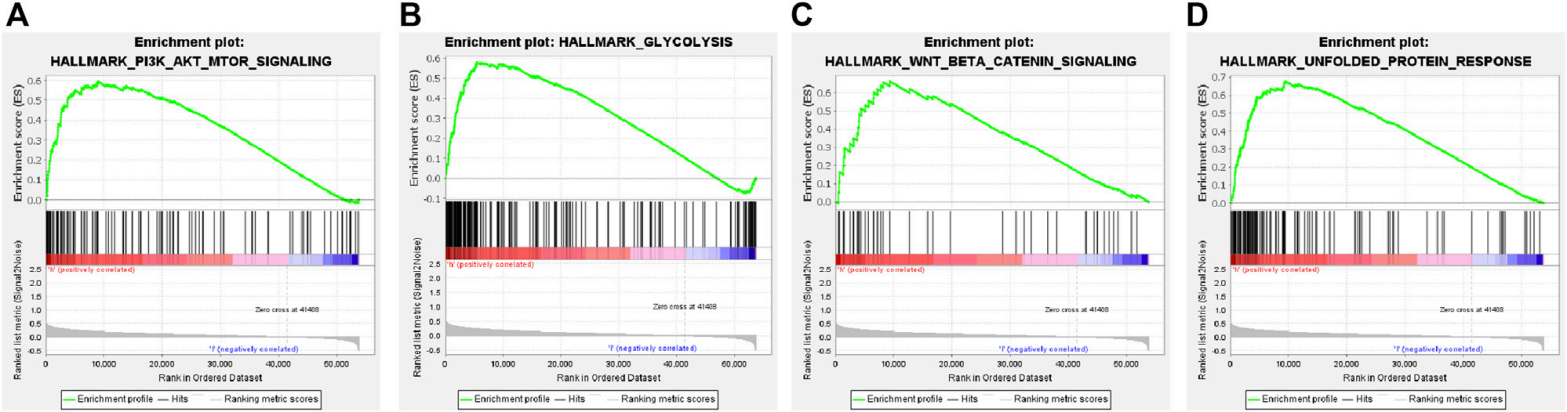
Gene set enrichment analysis of the cuproptosis-related ICG signature. **(A–D)** Four remarkably enriched tumor-associated HALLMARK pathways in the high-risk group.

The GSEA analysis revealed that the hallmark tumor-related pathways were primarily associated with the high-risk group, as shown in Supplementary Figure S4C. The pathways studied were PI3K-AKT-mTOR signaling, glycolysis, Wnt/beta-catenin signaling, and unfolded protein response pathways (Figures 8A–D). Bile acid and xenobiotic metabolism influenced the low-risk group (Supplementary Figure S4C).

A total of 637 DEGs were screened out. KEGG analysis revealed that the DEGs associated with the two groups were enriched in seventeen pathways (Supplementary Figure S4D, Supplementary Table S1), including glycolysis, cell cycle, tyrosine metabolism, and ECM-receptor interaction. Therefore, the findings demonstrated the biological significance of the prognostic gene signature.

### Analysis of the sensitivity of systemic therapy and immunotherapy

**FIGURE 9:**
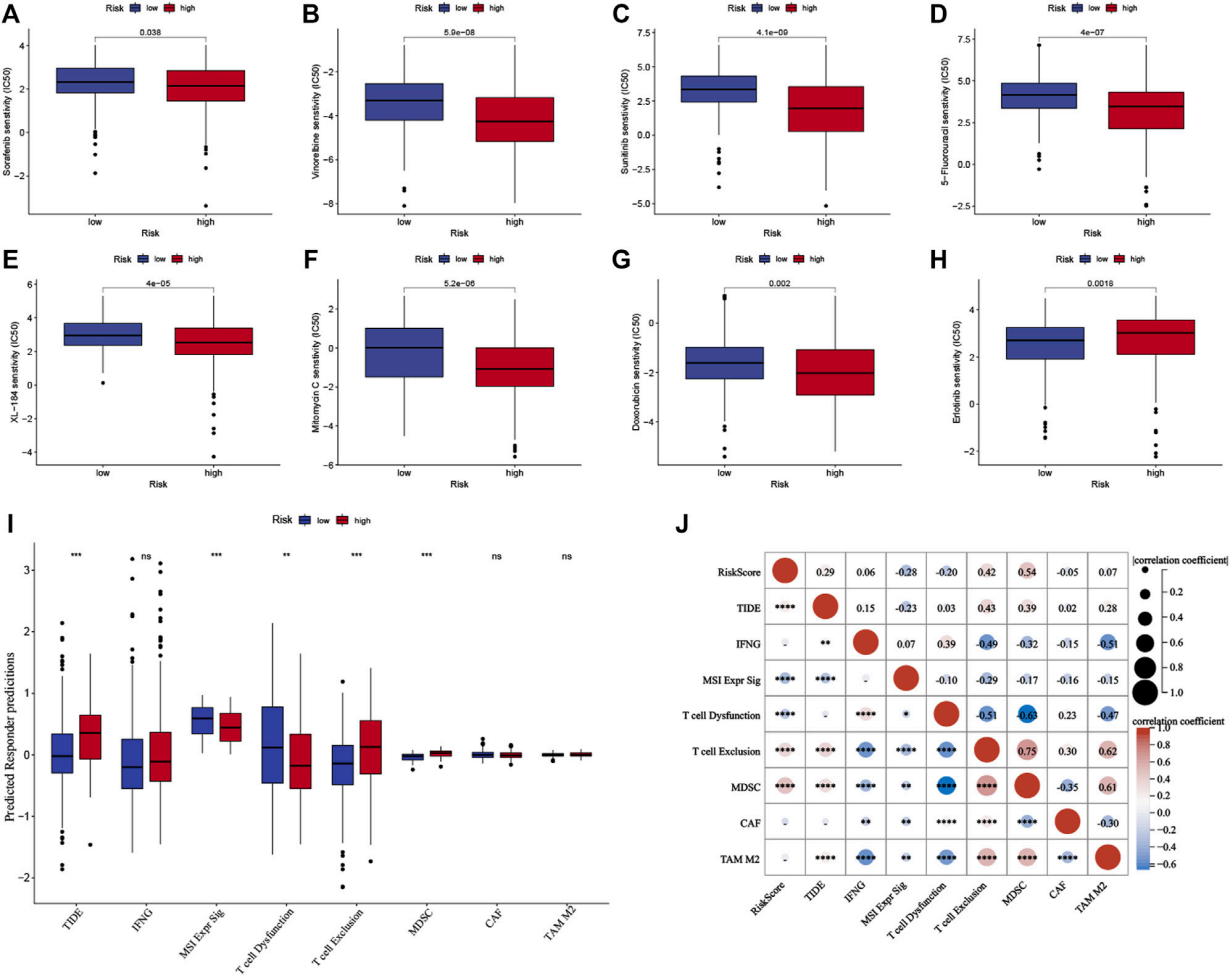
Predictive analysis of the prognostic signature for systemic therapy and immunotherapy in the TCGA cohort. **(A–H)** The half-maximal inhibitory concentration (IC50) of drugs for targeted therapy and chemotherapy in high- and low-risk groups in the TCGA cohort. **(I)** The Tumor Immune Dysfunction and Exclusion scores of high- and low-risk groups in the TCGA cohort. **(J)** Correlation analysis between risk score and TIDE score in the TCGA cohort. The upper and lower ends of the boxes indicate the interquartile range. Lines in the boxes indicate median values, and black dots show outliers. *p < 0.05; **p < 0.01; ***p < 0.001; ns, no statistical significance.

The half-maximal inhibitory concentration (IC50 value) of the first- and second-line targeted therapy methods was calculated using drug sensitivity analysis (Zhang et al., 2022). The most commonly used chemotherapeutics for HCC were also investigated. According to the findings, the IC50 value for erlotinib was lower in the low-risk group (*p* < 0.05, Figure 9H), whereas the IC50 values for sorafenib, vinorelbine, sunitinib, 5-fluorouracil, XL-184 (cabozantinib), mitomycin C, and doxorubicin were lower in the high-risk group (Figures 9A–G; *p* < 0.05) in the TCGA cohort. The ICGC cohort yielded comparable results (Supplementary Figures S5A–H).

TIDE score is calculated by assessing the T cell dysfunction and exclusion parameters, and it can predict tumor response to immune checkpoint inhibitors (ICIs). The TIDE scores were analyzed to determine the model’s accuracy in predicting the efficacy of immunotherapy. Figure 9I shows the TIDE scores for the groups, which indicated that high-risk group patients might have a poor response to immunotherapy (*p* < 0.001), and the results were confirmed for the ICGC cohort (*p* < 0.001, Supplementary Figure S5I). Furthermore, the characteristics of T cell exclusion and the myeloid-derived suppressor cells (MDSC) were analyzed, and the results are presented (*p* < 0.001, Figure 9I, Supplementary Figure S5I). Both cohorts had a positive correlation between the TIDE score, level of T cell exclusion realized, MDSC, and risk score (*p* < 0.01, Figure 9J, Supplementary Figure S5J).

Overall, the prognostic signature correlated with systemic therapy and immunotherapy response. This can aid scientists and clinicians in developing treatment methods based on patients’ sensitivity to targeted therapy and immunotherapy.

### Evaluation of genomic features and tumor mutation burden

The optimal cut-off value for classifying the samples into low- and high-TMB groups was determined. TMB levels were higher (*p* < 0.05) in the high-risk group than in the low-risk group (Supplementary Figure S6A). The survival rate in the low-TMB group was higher than in the high-TMB group (*p* < 0.05, Supplementary Figure S6B). A comprehensive survival analysis method was used for the risk score and TMB parameters (Supplementary Figure S6C), and patients with low TMB in the low-risk group had the best prognosis. The gene mutations were visualized as waterfall diagrams. Supplementary Figures S6D,E show that the low-risk group had a lower mutation rate (the top 15 genes) than the high-risk group. Both groups carried the missense mutation. The high-risk group had the highest *TP53* gene mutation frequency, while the low-risk group had the highest *CTNNB1* gene mutation frequency.

## Discussion

Most HCC patients are diagnosed at an advanced stage of the disease and thus do not benefit from radical treatment methods. Early diagnosis prolongs survival. Many patients go undiagnosed when they develop atypical symptoms at an early stage (Gou et al., 2019). Despite the promising results, only a few patients benefit from ICI-based immunotherapy. Under normal circumstances, the host immune system, mainly cytotoxic T lymphocytes (CTLs) and NK cells, can target and eliminate the malignantly transformed cells (Schumacher and Schreiber, 2015). However, the immune response is tightly regulated by a variety of activating and inhibiting mechanisms to prevent autoimmune events and maintain immune dynamic balance. As the main pathway to regulate the immune response, the immune checkpoints (ICs) signaling pathway will be activated, when the IC receptors, which are expressed on CTL and NK cells, interact with the IC ligands, which are expressed on tumor cells or immunosuppressive cells. At this time, the cytotoxicity and immune surveillance were suppressed, leading to tumor immune evasion (Kim et al., 2016). In addition, tumors can also restrain anti-tumor immunity by up-regulating the expression of ICs, resulting in an immunosuppressive tumor microenvironment (Wang et al., 2017). Cuproptosis-induced cell death has recently received much attention, but few studies have investigated the association between cuproptosis, prognosis, and anti-tumor immunity in HCC. The findings show that ICGs co-express with CRGs. A prognostic model based on five cuproptosis-related ICGs was established to investigate the relationship between gene expression signatures, prognosis, and anti-tumor immunity in HCC. Furthermore, the related signaling pathways were investigated, and the sensitivity to systematic therapy in patients with different expression signatures was assessed to better understand the differences in anti-tumor immunity in patients. Finally, the genetic variations were evaluated, including somatic mutations and their characteristics.

The TCGA-LIHC cohort was divided into two groups. The groups were established based on the expression levels of the five cuproptosis-related ICGs (*CD40LG*, *TNFRSF4*, *SIRPA*, *BTN2A1*, and *BTNL9*). We recorded poor prognoses for high-risk patients. The findings were confirmed for the ICGC cohort. Lower levels of expression of CD40LG, which is expressed on the CD4^+^ helper T cells as a co-stimulatory molecule, are observed in cancer patients, indicating an impaired immune response (Cai et al., 2021). Researchers have reported that CD40LG has a potent anti-tumor effect, which can be attributed to the CD40L–CD40LG interactions and can induce anti-tumor immunity by eliminating tumor-specific CD4^+^ and CD8^+^ tolerance (Sotomayor et al., 1999; Schmitz et al., 2001). TNFRSF4, a co-stimulatory receptor expressed by Tregs, can bind to OX40L and activate the NF-kB pathway (Song et al., 2008). Increased *TNRSF4* expression levels in HCC patients were associated with vascular invasion, high serum alpha-fetoprotein levels, and a poor prognosis (Xie et al., 2018). Pan et al. (Pan et al., 2013) indicated that SIRPA, an inhibitory molecule expressed by myeloid cells, is a crucial modulator in tumor-polarized macrophages. These could be potential therapeutic targets for HCC. The Vγ9Vδ2^+^ T cells are also promising anti-tumor therapy targets (Rigau et al., 2021). Cano et al. (Cano et al., 2021) reported that the *BTN2A1* gene influences Vγ9Vδ2^+^ T cells to mediate cytotoxic attacks on cancer cells. The anti-BTN2A1 monoclonal antibodies help to mitigate the cytotoxic effects of Vγ9Vδ2^+^ T cells on cancer cells (Cano et al., 2021). This indicates that the *BTN2A1* gene is a potential therapeutic target. The findings show that *LIPT1* and the signature genes co-express in significant amounts. HCC patients with high levels of *LIPT1* gene expression had a poor prognosis. *LIPTI* gene was also linked to immune cell infiltration. *LIPT1* gene encodes for liopyltransferase-1, which activates 2-ketoacid dehydrogenases involved in the tricarboxylic acid cycle (TCA cycle) (Ni et al., 2019; Solmonson et al., 2022). Liopyltransferase-1 transports the lipoic acid cofactor, including PD-ketoglutarate dehydrogenase, to the mitochondrial 2-ketoacid dehydrogenases involved in the TCA cycle (Solmonson et al., 2022). *LIPT1* gene is also involved in copper ionophore-induced cell death (Tsvetkov et al., 2022). LIPT1 deficiency can cause developmental delays, epilepsy, and broad metabolic abnormalities (Ni et al., 2019). However, the role of the *LIPT1* gene in cancer onset and progression remains unknown. The findings presented here shed light on the potential role of the *LIPT1* gene in cancer onset and progression. The findings may aid in developing novel ideas for future research.

The GSEA method investigated gene signatures, prognosis, and anti-tumor immunity mechanisms. Cancer cells experience an energy crisis as the extent of cell proliferation increases, forcing them to undergo metabolic reprogramming. (Dimri et al., 2020). Glycolysis is a critical metabolic pathway. It regulates proliferation, immune evasion, cell invasion, metastasis, angiogenesis, and drug resistance (Feng et al., 2020). Promoting the PI3K/AKT/mTOR pathway improves glucose transporter levels, increasing the rate of glycolysis, which promotes cancer progression (Dimri et al., 2020).

Stress from the abnormal accumulation of unfolded or misfolded proteins inside the endoplasmic reticulum (ER) significantly affects the progression of diseases such as cancer and diabetes. The unfolded protein response (UPR) pathway monitors the processes involved in endoplasmic reticulum protein homeostasis (Hetz et al., 2020). Oncogenic factors trigger ER stress and activate UPR, which can induce the process of oncogenic transformation and promote tumor growth, angiogenesis, and immune evasion (Hetz et al., 2020). UPR signaling can also control immune cell functions and differentiation. This method can also aid in establishing crosstalk with both adaptive and innate immune responses (Bettigole and Glimcher, 2015; Hetz et al., 2020). The activation of the Wnt/beta-catenin signaling pathway downregulates CCL5 expression and inhibits the DC recruitment process, resulting in increased resistance to ICIs and immune response escape in HCC patients (Ruiz de Galarreta et al., 2019). In addition, Wnt/beta-catenin signaling reduces the expression of the NKG2D ligand in HCC cells, hindering the generation of MHC-independent immune responses initiated by NK cells (Cadoux et al., 2021). These findings are consistent with our predicted results.

Complex molecular mechanisms drive anti-tumor immunity in HCC. The interaction of tumor cells with immune cells, and other immunomodulators in the tumor microenvironment determines the response to ICIs (Sia et al., 2017). The level of *LIPT1* gene expression correlated positively with the level of immune cell infiltration. This suggests a potential association between cuproptosis and immune infiltration levels. Tregs, Kupffer cells (which account for 90% of liver macrophages), monocyte-, and myeloid-derived macrophages have previously been identified as the key cells driving the immunosuppressive effect. In HCC patients, the functions of these cells result in the generation of evading immune responses. They also promote carcinogenesis and immune evasion *via* multiple mechanisms. The mechanisms include the secretion of various immunosuppressive cytokines and interleukin (IL)-10 and the recruitment of the Tregs cells and the CD4^+^ T helper 17 (Th17) cells (Llovet et al., 2022). Th2 cells, which primarily secrete IL-2 and IL-10, have been shown to promote immunosuppression, and tumor progression and metastasis. (Zhou et al., 2021). The results show a positive correlation between M0, Tregs, Th2 cells, follicular helper T cells, and the risk score, suggesting immunosuppression in high-risk group patients. These findings are also consistent with our prediction of the TIDE score.

Notably, it is crucial to explore the co-regulatory relationship between CRGs and ICGs at a deeper level, as this is the foundation of our successful clinical transformation. The advancement of single-cell multi-omics technologies is promising. Recent research has proposed a novel algorithm, the Single-cell Multi-omics Gene co-Regulatory algorithm (SMGR), (Song et al., 2022), which is efficient for identifying co-regulatory programs and is useful in determining molecular mechanisms and providing accurate targets. The single-cell multi-omics is the trend of molecular research in the future, and we look forward to more brand-new technologies that can inspire us.

There are a few limitations to the study. The findings presented here are based on bioinformatics analysis and lack experimental and clinical validation. Second, the reported direct relationship between cuproptosis, prognosis, and anti-tumor immunity in HCC patients need to be validated further.

## Conclusion

In conclusion, a novel cuproptosis-related ICG signature was developed for effective prognosis prediction beginning with ICGs that are co-expressed with CRGs. The immune response of HCC patients could also be predicted. Further research was conducted to explore the signaling pathways involved in the immune responses, cuproptosis, and level of immune infiltration. The findings presented here could aid in developing individualized treatment plans for HCC patients. It also contributes to a better understanding of the role of cuproptosis in patients’ prognosis and the development of anti-tumor immunity in HCC patients.

## Data Availability

The original contributions presented in the study are included in the article/Supplementary Material, further inquiries can be directed to the corresponding author.

## Supplementary material

Supplementary Figure S1
**(A)** The heatmap shows the differentially expressed cuproptosis-related genes (DECGs) between paracancerous and cancerous tissues in the TCGA cohort. **(B)** The Cytoscape visualizes the co-expressive relationship between DECGs and immune checkpoint genes.

Supplementary Figure S2
**(A–F)** Kaplan-Meier analysis of high- and low-risk groups in different subgroups of the ICGC cohort.

Supplementary Figure S3 Evaluation of the prognostic value of the signature. The receiver operating characteristic curves of the prognostic signature combined with the stages for **(A)** Predicting 1-, 3-, and 5-year survival rates of patients in the TCGA cohort and **(B)** predicting 1-, 2-, and 3-year survival rates of patients in the ICGC cohort. **(C)** The nomogram was constructed using the combination of risk score, age, gender, grade and stages. **(D)** The calibration curve of the nomogram. **(E)** The concordance index of risk score and other clinical parameters.

Supplementary Figure S4 Immune infiltration analysis, gene set and functional enrichment analysis of the prognostic signature. **(A)** and **(B)** Analysis of immune infiltration under different algorithms for TCGA and ICGC respectively. **(C)** The gene set enrichment analysis of high- and low-risk groups. **(D)** The Kyoto Encyclopedia of Genes and Genomes enrichment analysis of the signature.

Supplementary Figure S5 Predictive analysis of the prognostic signature for systemic therapy and immunotherapy in the ICGC cohort. **(A–H)** The half-maximal inhibitory concentration (IC50) of targeted therapy and chemotherapy drugs in the ICGC cohort’s high- and low-risk groups in the ICGC cohort. **(I)** The Tumor Immune Dysfunction and Exclusion (TIDE) score of the ICGC cohort’s high- and low-risk groups. **(J)** Correlation analysis between risk score and TIDE score in the ICGC cohort. The upper and lower ends of the boxes indicate the interquartile range. Lines in the boxes indicate median values, and black dots show outliers. **p* < 0.05; ***p* < 0.01; ****p* < 0.001; ns, no statistical significance.

Supplementary Figure S6 Evaluation of genomic features and tumor mutational burden (TMB). **(A)** TMB levels in high- and low-risk groups. **(B)** and **(C)** Kaplan-Meier analysis of patients with different TMB subgroups. The waterfall diagrams show the situation of gene mutations in the **(D)** high-risk group and **(E)** low-risk group.
