# Supplementary figures and images for: Cuproptosis-related immune checkpoint gene signature: Prediction of prognosis and immune response for hepatocellular carcinoma
Source: Front Genet. 2022 Oct 5;13:1000997. doi: 10.3389/fgene.2022.1000997 (PMC9579294; doi:10.3389/fgene.2022.1000997)

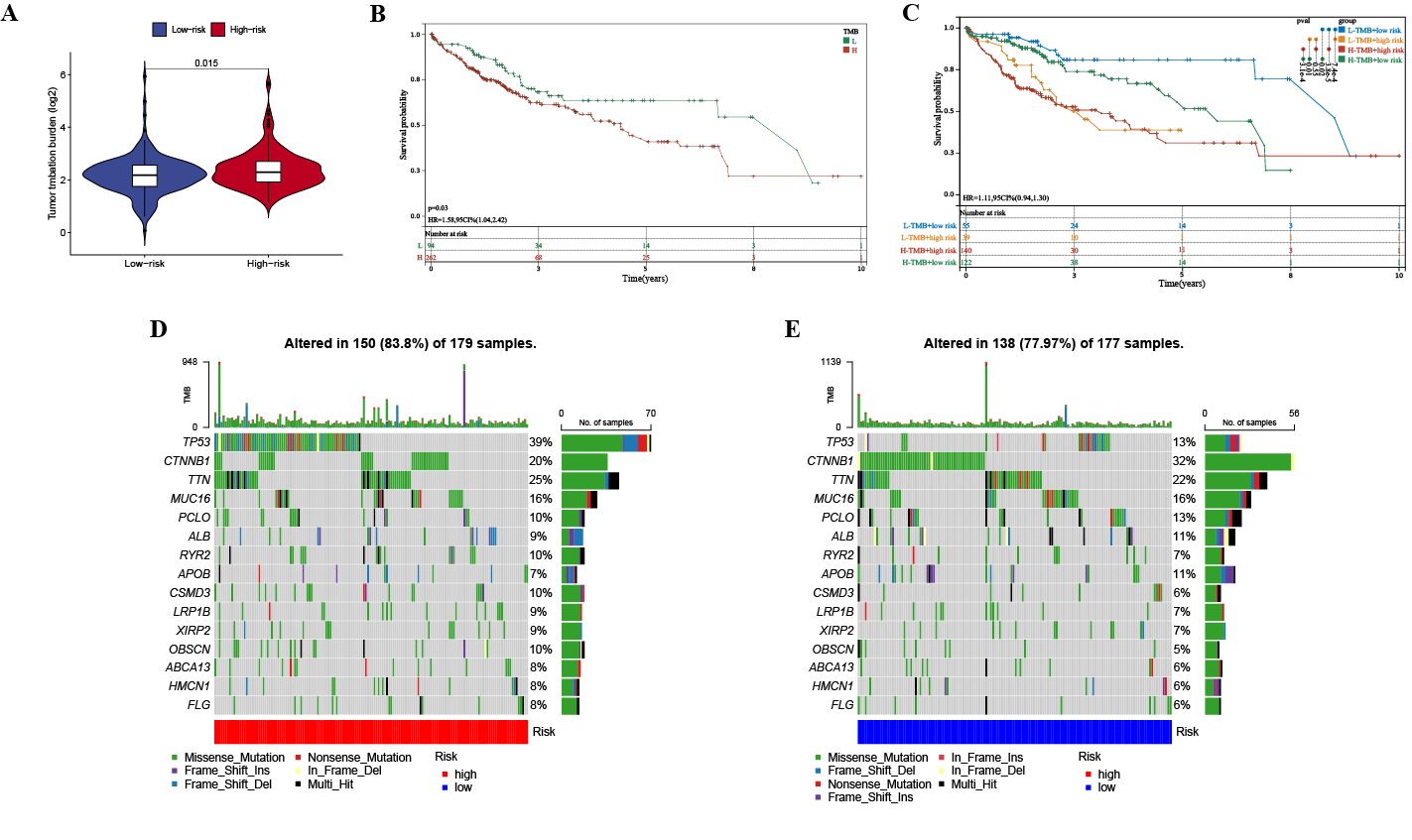

Supplement: Supplementary file 1 [file Image6.TIF]

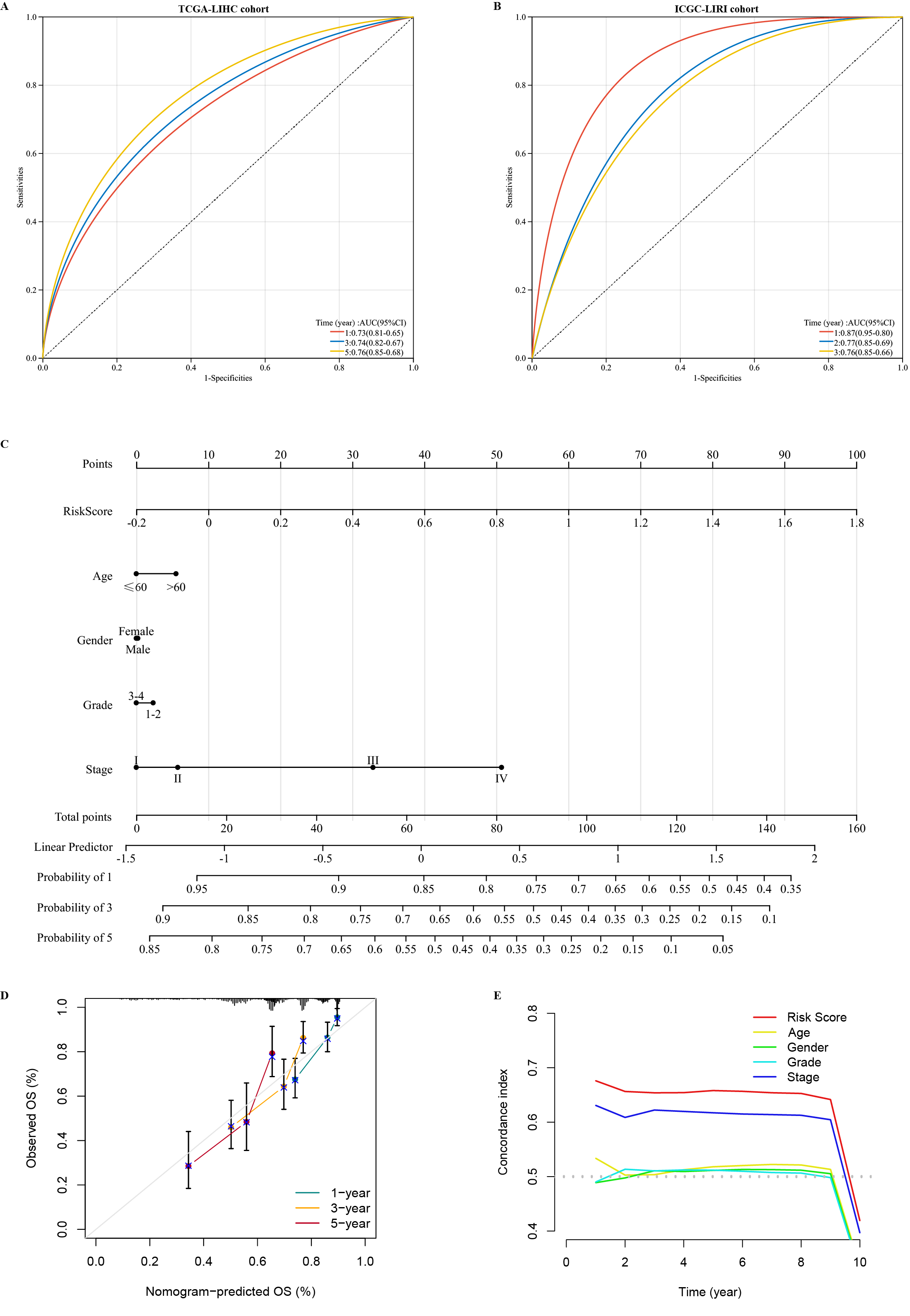

Supplement: Supplementary file 3 [file Image3.TIF]

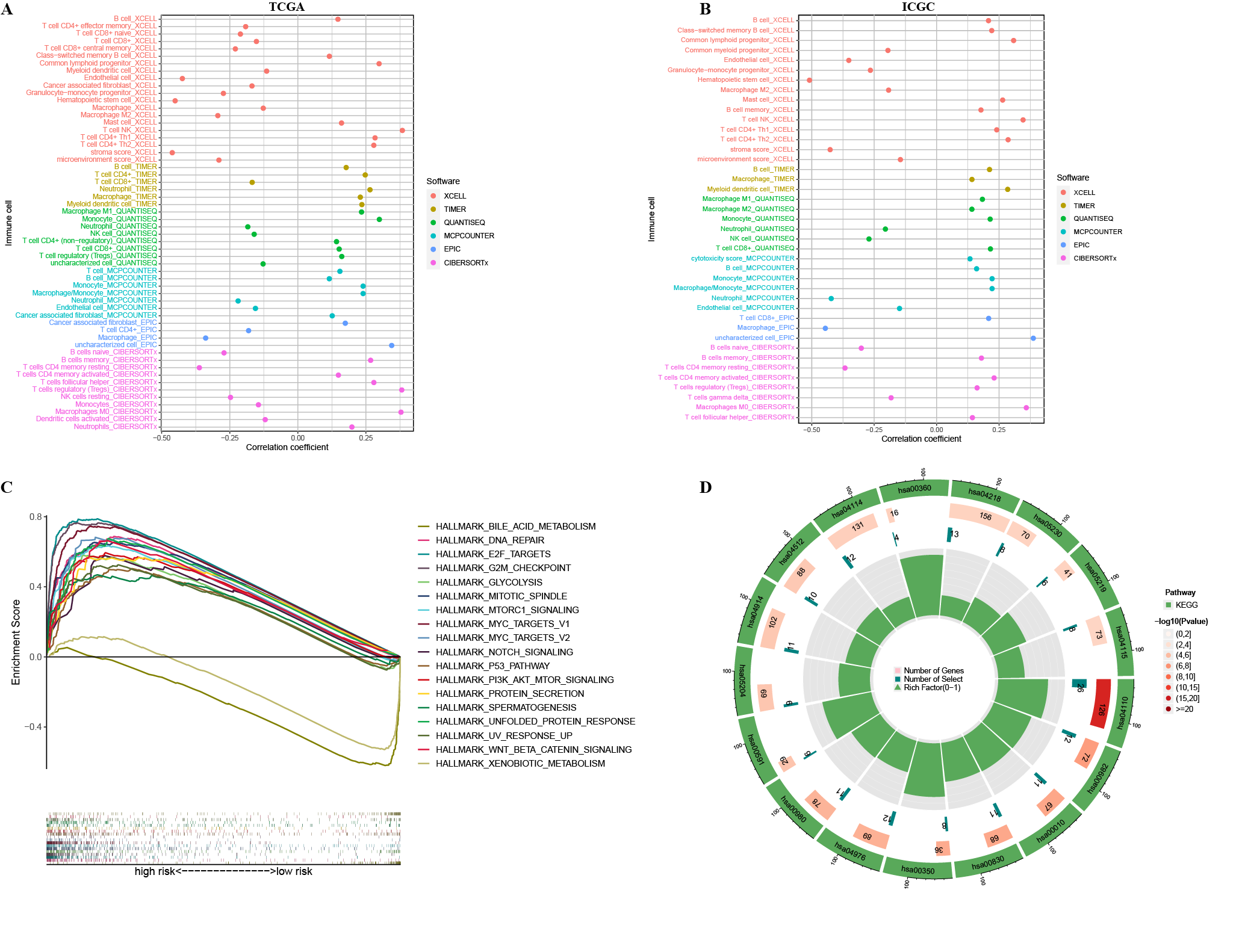

Supplement: Supplementary file 4 [file Image4.TIF]

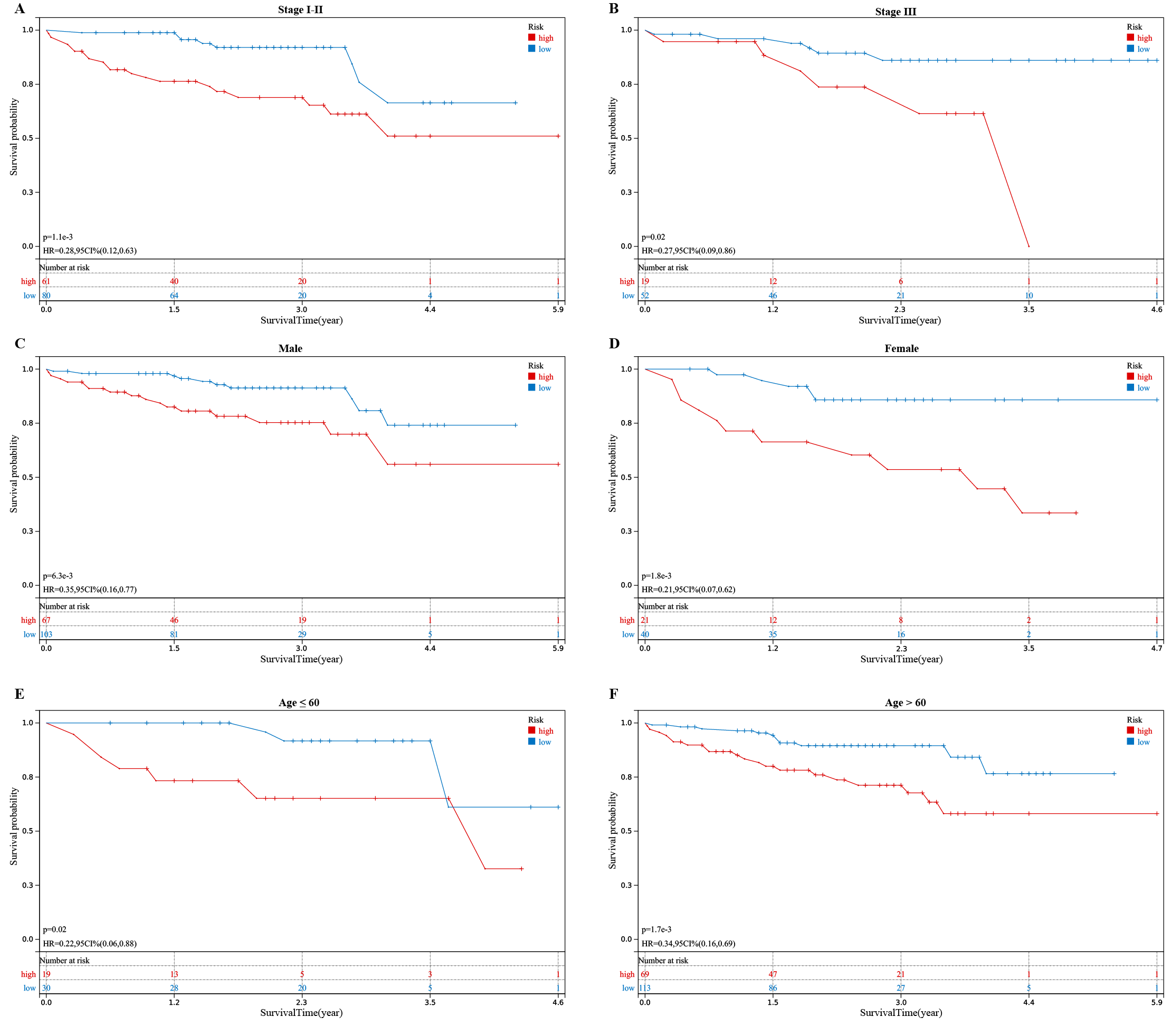

Supplement: Supplementary file 5 [file Image2.TIF]

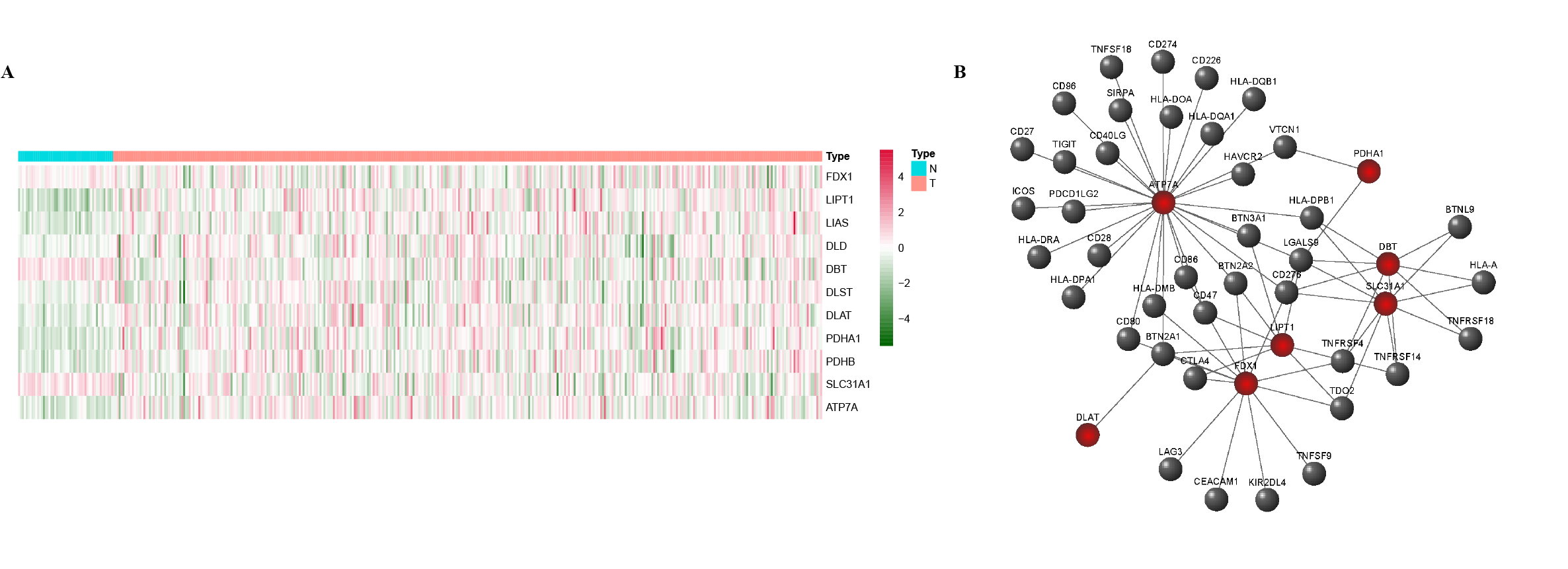

Supplement: Supplementary file 6 [file Image1.TIF]

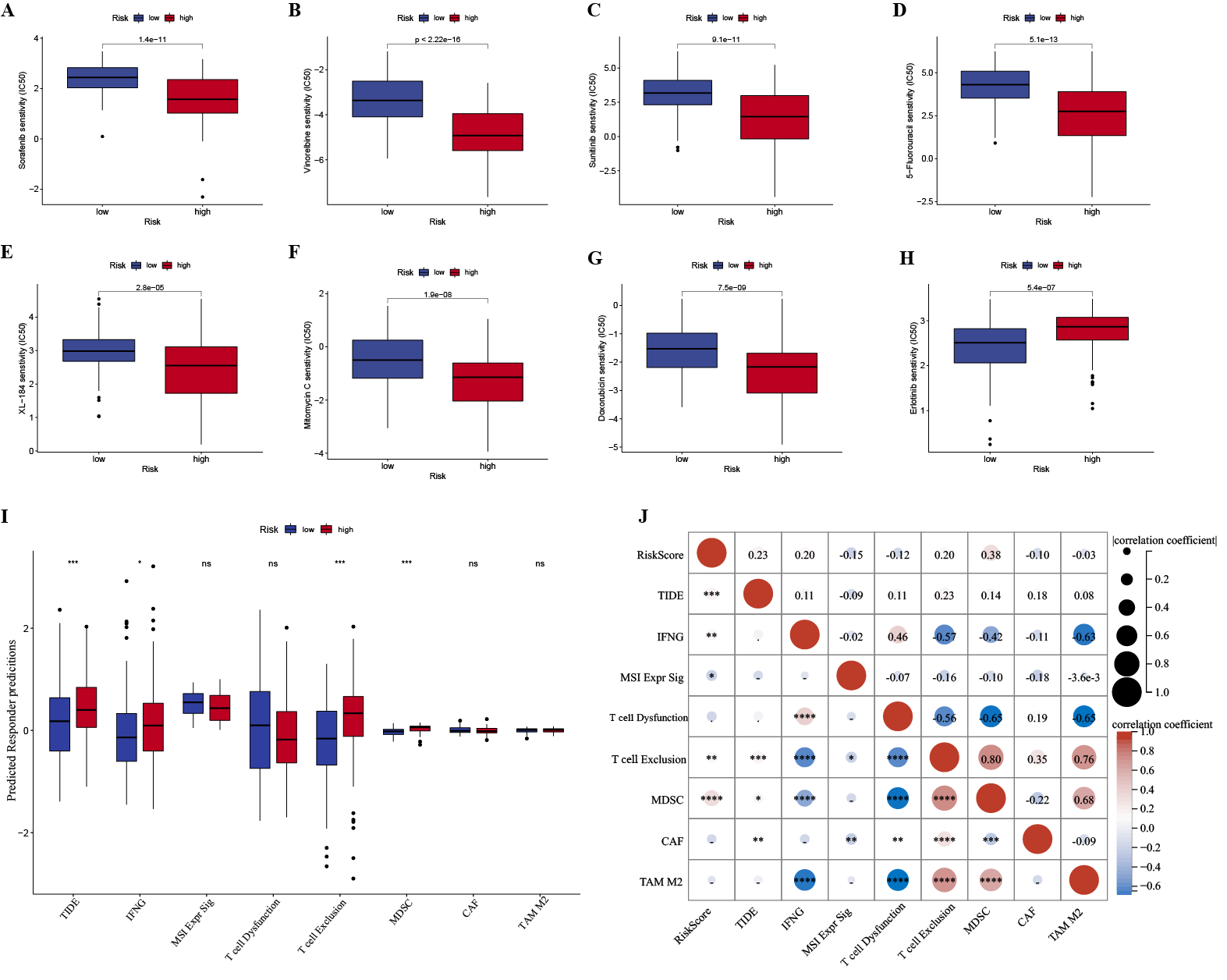

Supplement: Supplementary file 7 [file Image5.tif]
