# Supplementary material for: Cuproptosis-related immune checkpoint gene signature: Prediction of prognosis and immune response for hepatocellular carcinoma
Source: Front Genet. 2022 Oct 5;13:1000997. doi: 10.3389/fgene.2022.1000997 (PMC9579294; doi:10.3389/fgene.2022.1000997)
Supplement: Supplementary file 2 [file Table1.DOCX]

| Characteristics | TCGA cohort (n = 365) | | ICGC cohort (n = 231) | Total (n = 596) | *p-*value |
| --- | --- | --- | --- | --- | --- |
| Gender |  | |  |  | 0.13 |
| Female | 119 (19.97%) | | 61 (10.23%) | 180 (30.20%) |  |
| Male | 246 (41.28%) | | 170 (28.52%) | 416 (69.80%) |  |
| Age |  | |  |  |  |
| Mean ± SD | 59.65 ± 13.36 | | 67.30 ± 10.13 | 62.61 ± 12.76 |  |
| Median [min-max] | 61.00 [16.00, 90.00] | | 69.00 [31.00, 89.00] | 64.50 [16.00, 90.00] |  |
| Grade |  | |  |  |  |
| 1 | 55 (15.07%) |  | | 55 (15.07%) |  |
| 2 | 175 (47.95%) |  | | 175 (47.95%) |  |
| 3 | 118 (32.33%) |  | | 118 (32.33%) |  |
| 4 | 12 (3.29%) |  | | 12 (3.29%) |  |
| NA | 5 (1.37%) |  | | 5 (1.37%) |  |
| Stage |  |  | |  | < 0.05 |
| I | 170 (28.52%) | 36 (6.04%) | | 206(34.56%) |  |
| II | 84 (14.09%) | 105 (17.62%) | | 189 (31.71%) |  |
| III | 83 (13.93%) | 71 (11.91%) | | 154 (25.84%) |  |
| IV | 4 (0.67%) | 19 (3.19%) | | 23 (3.86%) |  |
| NA | 24 (4.03%) | 0 | | 24 (4.03%) |  |
| Survival Time (days) |  |  | |  |  |
| Mean ± SD | 811.93 ± 725.80 | 812.34 ± 418.56 | | 812.09 ± 624.50 |  |
| Median [min-max] | 596.00 [1.00, 3675.00] | 780.00 [10.00, 2160.00] | | 660.00 [1.00,3 675.00] |  |
| Survival Status |  |  | |  | < 0.05 |
| Alive | 235 (39.43%) | 189 (31.71%) | | 424 (71.14%) |  |
| Deceased | 130 (21.81%) | 42 (7.05%) | | 172 (28.86%) |  |

**Table 1.** Clinical baseline characteristics of the patients.

**Table 2.** Clinical baseline characteristics of patients with different risk groups in the TCGA cohort.

| Characteristics | Low-risk (n = 183) | High-risk (n = 182) | Total (n = 365) | *p*-value |
| --- | --- | --- | --- | --- |
| Age |  |  |  | 1 |
| > 60 | 96 (26.30%) | 96 (26.30%) | 192 (52.60%) |  |
| ≦ 60 | 87 (23.84%) | 86 (23.56%) | 173 (47.40%) |  |
| Gender |  |  |  | 0.11 |
| Female | 52 (14.25%) | 67 (18.36%) | 119 (32.60%) |  |
| Male | 131 (35.89%) | 115 (31.51%) | 246 (67.40%) |  |
| Grade |  |  |  | < 0.01 |
| Grade 1–2 | 133 (36.44%) | 97 (26.58%) | 230 (63.01%) |  |
| Grade 3–4 | 47 (12.88%) | 83 (22.74%) | 130 (35.62%) |  |
| NA | 3 (0.82%) | 2 (0.55%) | 5 (1.37%) |  |
| Stage |  |  |  | < 0.01 |
| I–II | 140 (38.36%) | 114 (31.23%) | 254 (69.59%) |  |
| III–IV | 33 (9.04%) | 54 (14.79%) | 87 (23.84%) |  |
| NA | 10 (2.74%) | 14 (3.84%) | 24 (6.58%) |  |

| Characteristics | Low-risk (n = 143) | High-risk (n = 88) | Total (n = 231) | *p*-value |
| --- | --- | --- | --- | --- |
| Gender |  |  |  | 0.59 |
| Female | 40 (17.32%) | 21 (9.09%) | 61 (26.41%) |  |
| Male | 103 (44.59%) | 67 (29.00%) | 170 (73.59%) |  |
| Age |  |  |  | 1 |
| > 60 | 113 (48.92%) | 69 (29.87%) | 182 (78.79%) |  |
| ≦60 | 30 (12.99%) | 19 (8.23%) | 49 (21.21%) |  |
| Stage |  |  |  | < 0.001 |
| I–II | 106 (45.89%) | 35 (15.15%) | 141 (61.04%) |  |
| III–IV | 37 (16.02%) | 53 (22.94%) | 90 (38.96%) |  |

**Table 3.** Clinical baseline characteristics of patients with different risk groups in the ICGC cohort.

**Table S1**. Kyoto Encyclopedia of Genes and Genomes analysis of enriched pathways

| ID | Description |
| --- | --- |
| hsa04110 | Cell cycle |
| hsa00982 | Drug metabolism-cytochrome P450 |
| hsa00010 | Glycolysis/Gluconeogenesis |
| hsa00830 | Retinol metabolism |
| hsa00350 | Tyrosine metabolism |
| hsa04976 | Bile secretion |
| hsa00980 | Metabolism of xenobiotics by cytochrome P450 |
| hsa00591 | Linoleic acid metabolism |
| hsa05204 | Chemical carcinogenesis-DNA adducts |
| hsa04914 | Progesterone-mediated oocyte maturation |
| hsa04512 | ECM-receptor interaction |
| hsa04114 | Oocyte meiosis |
| hsa00360 | Phenylalanine metabolism |
| hsa04218 | Cellular senescence |
| hsa05230 | Central carbon metabolism in cancer |
| hsa05219 | Bladder cancer |
| hsa04115 | p53 signaling pathway |
